# Trace elements fingerprint of feathers differs between breeding and non-breeding areas in an Afro-Palearctic migratory bird, the barn swallow (*Hirundo rustica*)

**DOI:** 10.1007/s11356-020-11597-z

**Published:** 2020-11-26

**Authors:** Marco Parolini, Michela Sturini, Federica Maraschi, Antonella Profumo, Alessandra Costanzo, Manuela Caprioli, Diego Rubolini, Roberto Ambrosini, Luca Canova

**Affiliations:** 1 Department of Environmental Science and Policy, University of Milan, via Celoria 26, I-20133 Milan, Italy; 2 Department of Chemistry, University of Pavia, via Taramelli 12, I-27100 Pavia, Italy

**Keywords:** Biomonitoring, Trace elements, Feathers, Barn swallow

## Abstract

Trace elements are widespread contaminants that can potentially threaten ecosystems and human health. Considering their distribution and toxicity, monitoring their presence in animals represents a priority in environmental risk assessment. Migratory birds have been suggested to be useful biomonitors for trace elements because they can provide information on contaminants even from remote areas that they may exploit during their life cycle. The aim of this study was to analyse the contamination fingerprint of trace elements of African non-breeding staging grounds and European breeding areas in a long-distance migratory passerine bird, the barn swallow (*Hirundo rustica*). We collected feathers grown in the African non-breeding grounds and those grown in the breeding areas of Northern Italy and measured the levels of 12 trace elements (Al, As, Cd, Cr, Cu, Fe, Hg, Mn, Ni, Pb, Se and Zn) by DRC-ICP-MS. Multivariate analysis showed that elemental profiles of feathers grown in African non-breeding areas and in the Italian breeding ones clearly differed, with feathers grown in Africa showing higher concentrations of Al, Cu, Fe, Mn and Ni, but lower concentrations of As, Se and Zn, compared to those grown in Italy. In addition, levels of trace elements were age-dependent, with higher levels in older individuals than in younger ones. Our results add to the growing evidence that feathers of long-distance migratory birds are useful tools to monitor trace elements contamination profiles across continents.

**Supplementary Information:**

The online version contains supplementary material available at 10.1007/s11356-020-11597-z.

## Introduction

The spread into the environment of a growing number of organic and inorganic contaminants due to human activities represents one of the main risks that all the living organisms have to face. Trace elements, in particular, have been recognized for a long time as an ubiquitous threat to the environment and human health (Abbasi et al. 2015). Trace elements are non-biodegradable, globally distributed contaminants originating by natural geological and diverse anthropogenic processes (Burger and Gochfeld 1991). Although some trace elements play an essential role in biological functions (e.g. zinc, copper and iron) or may have positive effects even in small quantities (e.g. manganese or nickel), others have no biological role and may exert toxic effects or even be potentially fatal also in low amounts (e.g. cadmium, lead or arsenic; Aras and Ataman 2007). Moreover, some trace elements can accumulate along the trophic chain (Burger and Gochfeld 2004; Bostan et al. 2007) and induce deleterious effects across multiple levels of the ecological hierarchy. In fact, the accumulation of trace elements can be a major environmental stressor for wild animal species (Lattin et al. 2014; Romero and Wikelski 2001; Wikelski et al. 2002). Trace elements are known to induce physiological responses resulting in negative fitness effects (Cyr and Romero 2007; Romero 2004), ultimately impacting on population dynamics (Wikelski and Cooke 2006). In birds, exposure to trace elements can induce physiological alterations leading to negative consequences for eggs, immune system functioning, post-natal growth and reproduction (Jackson et al. 2011; Nighat et al. 2013). Thus, considering their widespread distribution and toxicity, it is pivotal to monitor the levels of trace elements in animals (Innangi et al. 2019). Because of their peculiar ecological features, birds have been identified for a long time as useful indicators of trace elements contamination (Hahn et al. 1993; Denneman and Douben 1993). Birds are ubiquitous, generally easy to sample and can be used as indicators either of long-range or local pollution depending on their migratory behaviour (Baker et al. 2017; Borghesi et al. 2016; Bortolotti 2010). Moreover, birds use different sources of food and water in relatively large areas so that the levels of trace elements measured in their organs and/or feathers might reveal the contamination levels in their whole home range. Lastly, a number of bird species show a high philopatry. Species living in close association with human activities are exposed to anthropogenic contaminants and may experience contaminant-induced toxic effects (Malik and Zeb 2009). For this reason, birds can be used as ‘sentinels’ for potentially negative consequences of trace elements for human health.

Levels of trace elements in birds can be measured in different organs, including the liver, muscle or kidneys (Cosson et al. 1988; Goutner et al. 2011; Taggart et al. 2009). However, non-invasive procedures relying on the collection of feathers (or excrements) represent a successful non-lethal approach (e.g., Costa et al. 2013). In particular, bird feathers represent an excellent biological matrix to assess levels of different environmental contaminants, including trace elements, because they can integrate contamination patterns across broad spatial and temporal scales (Rutkowska et al. 2018). Specifically, trace elements can bind to protein molecules during the period of feather growth, when feathers are connected with the bloodstream through small blood vessels (Burger 1993). At the end of feather growth, the blood vessels experience atrophy, and the feather remains physiologically isolated from the bird (Denneman and Douben 1993). The moult of feathers represents a pathway used by birds to eliminate a great portion of their body burden of trace element through their plumage (Burger 1993; Hughes et al. 1997). During the moult, the levels of trace elements measured in the bloodstream of diverse bird species drop because they are sequestered in the feathers (Dauwe et al. 2003). Thus, the levels of trace elements measured in feathers accurately reflect the body burden of these contaminants during feather formation. Provided that the timing and geographical location where feathers are grown is approximately known, feathers can thus represent useful biological ‘archives’ of environmental contamination levels. Although a number of studies have monitored the levels of trace elements using feathers of resident bird species, the information for migratory species is still limited. To date, the levels of trace elements were measured in tail feathers of two insectivorous and long-distance migratory bird species, the sand martin *Riparia riparia* (Szép et al. 2003, 2009) and the barn swallow *Hirundo rustica* (Szép et al. 2009) in order to identify moulting areas and migratory pathways, as well as in feathers of the collared flycatcher *Ficedula albicollis* (Óvári et al. 2018) and the great crested flycatchers *Myiarchus crinitus* (Cooper et al. 2017).

The present study aimed at investigating the presence of trace elements in feathers of the European barn swallow *Hirundo rustica* subsp. *rustica*, a small, long-distance migratory passerine bird that breeds in Eurasia and spends the non-breeding season in the Afrotropics (Turner 2006). The levels of 12 trace elements (Al, As, Cd, Cr, Cu, Fe, Hg, Mn, Pb, Ni, Se, Zn) accumulated in feathers were related to the age and the sex of the bird and to the geographical areas where the feather were grown. Specifically, we explored the potential differences between trace element levels accumulated in barn swallow feathers grown in the African non-breeding areas and in those grown in the area of Northern Italy where the sampled individuals bred. Concentrations of trace elements in feathers grown in Africa represented the levels accumulated in the non-breeding quarters where barn swallows undergo their annual complete moult, whereas those grown in Italy reflected the contamination level of the breeding quarters. In addition, age and sex differences in trace element fingerprint, as well as the covariation between some phenotypic traits (i.e. body mass and wing chord) and trace element levels were investigated. Despite we have no evidence of different habitat selection or non-breeding distribution between male and female or between 1-year-old or older individuals (Liechti et al. 2015), we cannot exclude a priori a different exposure to contaminants and/or physiological differences in contaminant accumulation between sexes or age classes in breeding and non-breeding areas.

## Materials and methods

### General methods and sampling of feathers

The European barn swallow is a small (ca. 15 g) trans-Saharan migratory passerine bird, which undergoes a single, complete annual moult in sub-Saharan wintering quarters (Cramp 1998). Between April 30 and July 8, 2012, adult barn swallows breeding at five different colonies (i.e. farms) in Northern Italy were captured using mist nets (centroid of the study area: 45° 36′ N, 8° 37′ E). Each individual was ringed and sexed according to the presence (females) or absence (males) of the brood patch (Møller 1994; Turner 2006). Individual age was known with precision thanks to intensive ringing of individuals conducted at the same colonies in previous years and to the extreme breeding site fidelity of this species, which allow assuming that individuals captured for the first time in a given colony are 1-year-old (see Saino et al. (2002) for details on this method for ageing barn swallows). Body mass was measured with a Pesola spring balance to the nearest 0.1 g, and the wing chord was measured on the unflattened right wing with a ruler to the nearest mm. As some previous studies showed that the levels of trace elements can vary among the type of feathers (e.g. Dauwe et al. 2003), we focused on the fourth (counting outwards) right rectrix (R4) only. The R4 was plucked and stored in sealed plastic bags to avoid contamination during collection and transportation to the laboratory. This feather was grown in the African non-breeding areas (hereafter, R4nb), as barn swallows complete their annual moult before starting their migration towards the breeding quarters. Thus, trace element levels in this feather represented the contamination profile of the African non-breeding areas of the study population, located in an equatorial area 1000 km in radius, centred in Cameroon, as shown by a geolocator tracking study (Liechti et al. 2015). Due to their importance during flight, birds can facultatively re-grow feathers that are accidentally lost due to, e.g. predation attempts or territorial disputes (Møller et al. 2006). Hence, the removal of R4 induced a regrowth of the same feather, which is completed in ca. 40 days after the R4 removal in both sexes (Saino et al. 2014). Thus, about 1 month after the first capture (mean difference between the first and the second capture in days: 29.1 ± 4.9 SD), the same adults were re-captured; the re-grown R4 was plucked and stored as described before until chemical analyses. The trace element levels in the re-grown R4 thus represented the contamination profile of the breeding areas (hereafter, R4br). A total of 59 adults, 32 males and 27 females, 39 1-year-old, 17 2-year-old and 3 3-year-old were captured during the sampling survey, and their tail feathers (for a total of 118 feathers) were analysed for elemental composition. Because of small sample size of 3-year-old individuals, they were grouped with 2-year-old ones. Thus 20 > 1 year-old individuals were included in the analyses.

### Reagents

Trace-SELECT® Ultra ultrapure HNO_3_ (65% w/w), H_2_O_2_ (30% w/w) and certified multi-standard solution Merck VI for ICP-MS were supplied by Sigma Aldrich (Milan, Italy). The multi-element standard solutions were daily prepared in 0.5% ultrapure nitric acid by diluting ICP stock solution. Ultrapure water was produced in laboratory by a Millipore Milli-Q system.

### Apparatus

The samples microwave digestion was performed by a CEM Mars microwave oven (Mars 5, CEM s.r.l., Cologno al Serio, Italy) equipped with eight PFA PTFE (PerFluoroAlkoxy PolyTetraFluoroEthylene) (Xpress, 55 ml) vessels, providing 1600 W output power at 100% power setting, and an internal IR temperature sensor for the temperature control. Evaporation of the digested acid solutions was carried out by XpressVapTM accessory. Measurements were performed by an inductively coupled plasma quadrupole mass spectrometer (ICP-MS) (Elan DRC-e, PerkineElmer, Shelton, CT, USA) equipped with a standard ICP torch, cross-flow nebulizer, nickel sampler and skimmer cones and dynamic reaction cell™ (DRC).

### Analytical procedure

Before the dissolution step, the feathers were vigorously washed in deionized water, sonicated for 5 min in acetone diluted with ultrapure water (about 1 M), rinsed in ultrapure water and then air-dried, according to the procedure reported in Ansara-Ross et al. (2013). Each feather was accurately weighed into the PFA PTFE vessels of the microwave digestion system (ca. 10 mg each), and 5 ml of HNO_3_ plus 2 ml of H_2_O_2_ were added. The whole feather from each individual was processed. Microwave heating was then performed at 1600 W for 15 min (ca. 200 °C as measured by an internal IR temperature sensor for the temperature control). The digestion led to a complete dissolution; therefore, filtration was not necessary. After cooling, the obtained clear solutions were evaporated to a small volume (ca. 0.5 ml), diluted to 5 ml with ultrapure water in calibrated polypropylene tubes and analysed by DRC-ICP-MS for trace elements determination. Three-point calibration curves were generated in the range 5–500 μg/l. Method detection and quantification limits (MDLs, MQLs, respectively) were obtained from the instrumental detection and quantification limits (IDLs, IQLs, respectively) calculated using the residual standard deviation (Sy/x) of the linear regression parameters as (3.3 × Sy/x)/slope and (10 × Sy/x)/slope, respectively, and are referred to the overall procedure (See Table S1). Reagent blanks were prepared following the same procedure applied to samples. Five sets of method blanks and certified reference material (BCR-397, trace elements in human hair) were processed and analysed concurrently with samples. The data obtained are reported on a dry weight basis (mg/kg dry weight).

### Statistical methods

Trace element concentrations were compared between R4nb and R4br (i.e. feather of the same individual grown in Africa and Italy, respectively), as well as between individuals according to sex and age using both univariate and multivariate models.

For univariate analyses, we relied on linear mixed models where concentration of each element in a feather was modelled according to whether the feather was grown in Africa or in Italy (feather growth location), the sex of the individual, its age (i.e. whether an individual was 1-year-old or older) and the interaction between feather growth location and the other predictors. To account for non-independence of data (i.e. the fact that we collected two feathers from the same individual and that different individuals were captured in the same farm), farm and individual identity were entered as random grouping factors, and feather growth location (dichotomic factor) was entered as a random slope within individual. This parameterization allowed making pairwise comparisons between the feathers collected on the same individual (Zuur et al. 2009). Models also accounted for heterogeneity of variance between groups identified by feather growth locality and individual age. Since we detected potential deviation from model assumptions, we further checked the significance of these models through a randomization procedure (9999 repetitions) that accounted for the grouping structure of the data (see Supporting Information for details). The use of a randomization procedure to assess significance in these linear models assures the robustness of the results to possible deviations from linear model assumptions (Sokal and Rohlf 2011). Since we were performing a large number of statistical tests when comparing the concentrations of multiple elements in the same feather, we avoided inflating type I error rate by correcting *P* values with the Benjamini-Hochberg method (Benjamini and Hochberg 1995).

For multivariate analyses, we relied on redundancy analyses (RDA) whereby concentration of all elements was regressed simultaneously on feather growth location, sex, age and the interactions between feather growth location and the other predictors. RDA can be considered a multivariate extension of analysis of variance as well as an extension of principal component analysis whereby axes are linear combinations of predictors (Legendre and Legendre 2012). Concentrations of each element were standardized before the analysis by subtracting the mean value and dividing for the standard deviation to reduce the importance of elements whose concentrations showed the largest variances. We also repeated the analyses by including in separate RDA models either body mass or wing chord to investigate whether levels of trace elements were related to phenotypic traits of barn swallows. Significance of RDA models was assessed through the same randomization procedure used for univariate analyses (see Supporting Information for details). Finally, the patterns of associations among trace element concentrations in feathers were investigated by calculating the Pearson correlation matrices among element concentrations separately for feathers grown in Africa and Italy. These correlation matrices were visualized through heatmaps.

Analyses were performed with the R statistical software version 3.6.2 (R Core Team 2019) with the vegan (Oksanen et al. 2019), glmmTMB (Brooks et al. 2017), emmeans (Russell 2020), multtest (Pollard et al. 2005), and sjPlot (Lüdecke 2020) packages.

## Results and discussion

Our results showed that the elemental profile of barn swallow feathers differed between African non-breeding and Italian breeding areas, with specific patterns depending on the geographical origin of feathers. Moreover, trace element levels significantly differed depending on individual age, with higher levels measured in feathers from older individuals compared to younger ones.

### Levels of trace elements in barn swallow feathers

Focal trace elements were detected in tail feathers from all individuals, both in R4nb and R4br, with the exception of Cd, which was found in measurable concentrations only in 44% of samples. Overall, the most abundant trace elements were Al, Fe and Zn, with an average concentration (± SE) of 221 ± 24 mg/kg, 148 ± 2.5 mg/kg and 146 ± 6 mg/kg, respectively, independently of the individual age, sex and feather type (R4nb or R4br). In contrast, the levels of all the other trace elements were lower than ~ 11 mg/kg, with As and Cd found in negligible amount (Table 1). The concentrations of Al, the most abundant trace element found in barn swallow feathers, were three times larger among R4nb compared to R4br (Table 1). This is probably because barn swallows from our breeding population spread across a broad African non-breeding range, spanning thousands of kilometres (Liechti et al. 2015), and trace elements of R4nb thus reflect the contamination profile of areas characterized by different elemental compositions. In contrast, the narrow variability of trace elements in R4br was expected as all feathers were grown within the same small area (~ 20 km in radius).

**Table 1:** Mean, standard error (SE), minimum (min) and maximum (max) value of the 12 trace elements measured in feathers grown in non-breeding staging and breeding grounds by 1-year-old or older barn swallows

	1-year-old				> 1-year-old			
	Mean	SE	Min	Max	Mean	SE	Min	Max
Non-breeding staging grounds								
Al	218.88	31.48	71.27	1011.55	533.75	94.01	131.07	1553.56
Fe	154.58	6.82	75.20	294.91	246.25	20.87	126.39	447.96
Zn	133.85	2.95	87.17	196.21	143.94	4.75	118.58	200.05
Cu	10.86	0.42	7.25	21.93	11.45	0.52	8.42	16.28
Mn	8.97	0.45	2.75	16.09	12.60	1.20	5.54	25.21
Ni	4.99	0.27	2.89	12.82	4.74	0.38	3.05	9.58
Pb	4.12	0.26	1.96	8.87	3.27	0.29	1.94	6.17
Hg	2.92	0.39	0.00	13.77	3.74	0.73	0.32	12.55
Se	1.35	0.13	0.21	3.92	1.35	0.28	0.36	4.55
Cr	1.02	0.07	0.42	2.32	1.29	0.11	0.76	2.39
As	0.37	0.01	0.18	0.54	0.33	0.03	0.18	0.53
Cd	0.02	0.01	0.00	0.13	0.05	0.01	0.00	0.13
Breeding grounds								
Al	115.75	11.72	36.35	400.49	154.86	26.60	40.80	496.23
Fe	102.48	5.47	57.32	182.95	136.79	6.66	99.36	196.16
Zn	160.91	5.85	111.08	361.35	141.53	3.48	116.05	175.09
Cu	9.40	0.44	5.68	20.85	9.20	0.56	5.68	14.15
Mn	2.04	0.21	0.64	6.47	3.73	0.69	0.69	14.16
Ni	4.18	0.22	2.98	9.99	4.08	0.17	2.98	5.69
Pb	3.91	0.23	1.84	7.45	4.06	1.10	1.26	21.51
Hg	2.16	0.14	0.55	3.84	2.23	0.21	0.70	4.11
Se	2.10	0.15	0.74	5.11	2.24	0.27	1.01	4.86
Cr	0.99	0.14	0.29	5.12	1.01	0.20	0.37	3.53
As	0.43	0.02	0.22	0.68	0.34	0.03	0.15	0.62
Cd	0.03	0.01	0.00	0.18	0.07	0.01	0.00	0.14

All values are expressed as mg/kg

The average concentration of trace elements accumulated in tail feathers of barn swallows decreased as follows: Al > Fe > Zn > Cu > Mn > Ni > Pb > Hg > Se > Cr > As > Cd. The levels and the elemental fingerprint observed in the present study were similar to those measured in feathers of diverse passerine species breeding in other geographical areas, including great tits *Parus major* from Belgium (Veerle et al. 2004), collared flycatchers from Hungary (Óvári et al. 2018), sand martins from different European breeding areas (Vallner et al. 2000,; Szép et al. 2003) and Italian sparrows *Passer italiae* from southern Italy (Innangi et al. 2019), while they differed compared to those measured in tail feathers collected from house sparrows *Passer domesticus* living in southern Africa (Baker et al. 2017). The toxic elements included in the priority list of contaminants of 2013/39/EU directive for surface water, namely Cd, Hg, Ni and Pb, were above the detection limit in all samples, with the exception of Cd. As reported for other elements, the levels of Hg, Ni and Pb were similar to those reported for feathers of song sparrows *Melospiza melodia* from upper Santa Cruz River in Arizona (Lester and van Riper 2014), collared flycatchers from Hungary (Óvári et al. 2018) and cattle egrets *Bubulcus ibis* from Pakistan (Abdullah et al. 2015).

### Age- and geographical-specific differences in trace element levels

**Fig. 1:**
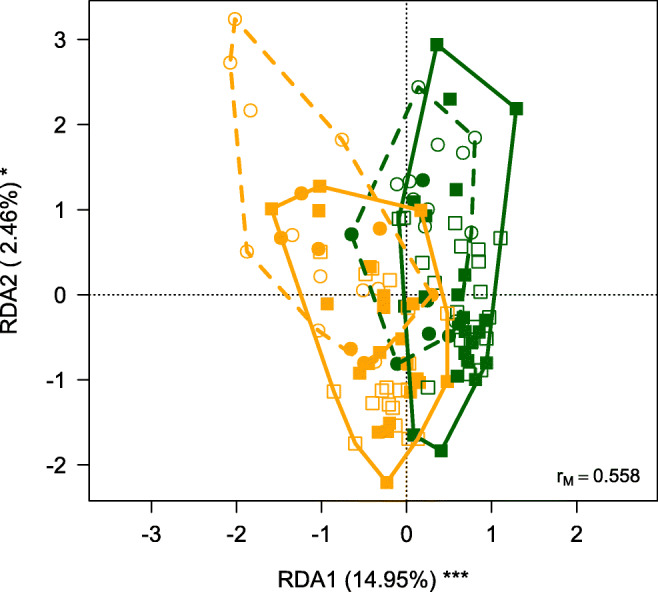
Biplot from the redundancy analysis of standardized element concentrations in barn swallow feathers on locality of feather growth, sex, age and the interactions between locality of feather growth and the other predictors. Symbols denote feathers. Squares represent 1-year-old individuals, circles represent older individuals, open symbols represent males and filled symbol females. Yellow symbols denote feathers grown in Africa, green symbols feathers grown in Italy. Contours include feathers grown in Africa by 1-year-old individuals (yellow solid line line) or by older individuals (yellow dashed line), and feathers grown in Italy by 1-year-old individuals (green solid line) or by older individuals (green dashed line). Variance explained by the first two axes is reported as well as axis significance ***P < 0.001; *P < 0.05). *r*_M_ is the Mantel correlation coefficient between Euclidean distances among samples in the multivariate space and Euclidean distances among symbols representing each sample in the plot. Values close to one indicate that the plot accurately represents distances among samples

The RDA analysis showed that elemental composition differed significantly between R4nb and R4br and between 1-year-old and older individuals (Table 2; Fig. 1). In addition, also the interaction between feather growth location and individual age was significant (Table 2). In contrast, the levels of trace elements were not related to body mass or wing chord of barn swallows surveyed in this study, both when we considered R4nb (RDA: F_1,57_ ≤ 1.573, *P* ≥ 0.225) or R4br (RDA: F_1,57_ ≤ 1.546, *P* ≥ 0.143), suggesting that body condition was not related to the accumulation of trace elements in both wintering and breeding areas. We therefore discarded body mass and wing chord from univariate analyses.

**Table 2:** Results from redundancy analysis on standardized abundance of contaminants in feathers according to locality of feather growth, barn swallow sex and age and the interactions between locality of feather growth and the other predictors

Variable	df	Variance	*F*	*P*
Locality	1	0.923	10.896	*< 0.001*
Sex	1	0.092	1.087	0.323
Age	1	0.576	6.804	*0.001*
Locality × sex	1	0.084	0.995	0.288
Locality × age	1	0.212	2.501	*0.007*
Residuals	112	9.487		

Significant effects are reported in italic

Adjusted *R*^2^ = 0.174

**Fig. 2:**
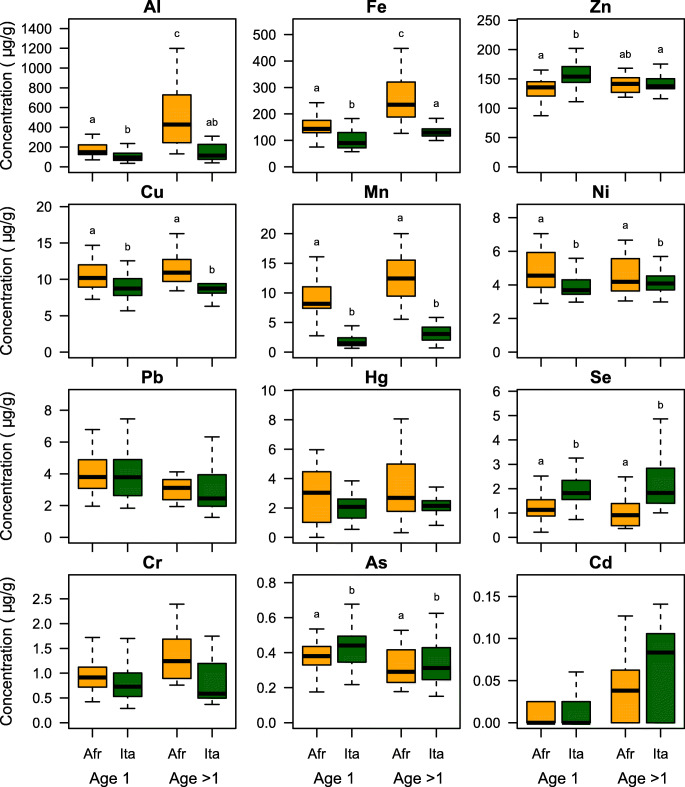
Concentrations of trace elements in tail feathers grown in non-breeding staging (Afr) or breeding grounds (Ita) of 1-year-old (age 1) or older barn swallows (age > 1)

Differences in the overall elemental composition of feathers were further investigated with univariate analyses (Tables 3 and S2) that showed that, in particular, R4nb had higher concentrations of Cu, Mn, and Ni than R4br (Fig. 2). In contrast, Se and As concentrations were higher in R4br than in R4nb (Fig. 2). Mn concentrations were also lower in 1-year-old individuals than in older ones (Fig. 2). The interaction between feather growth location and age was significant in the models of Al, Fe and Zn, the three most abundant elements. For Al and Fe, R4nb showed lower concentrations than R4br, and this difference was much larger for older individuals than for 1-year-old ones. Generally, the highest concentrations of both elements were measured in R4nb by > 1-year-old individuals. An opposite pattern was observed for Zn, with R4br showing higher concentrations than R4nb, and the highest values observed in R4br by 1-year-old individuals. No significant difference (after FDR correction) was observed in Pb, Hg, Cr and Cd concentrations (Tables 3 and S2; Fig. 2). The differences in the profile of trace elements accumulated in barn swallow tail feathers grown in Africa and Italy suggest that the elemental composition depended largely on the micro-geographical position of the areas where birds grew their feathers. Moreover, such differences may reflect a dissimilar elemental profile of soil, water, vegetation and/or food sources occurring between the wintering grounds and the breeding areas. Higher levels of trace elements in old than young individuals were already observed in non-passerine birds. For instance, Fe, Cu and Pb accumulated with age in kestrels (Kim and Oh 2017), while, to the best of our knowledge, this is the first study reporting this effect in passerines.

**Table 3:** *P* values corrected with the false discovery rate procedure (*P*_FDR_) of univariate analyses of elemental abundance

	Location	Sex	Age	Location × sex	Location × age
Al	*0.035*	0.912	0.063	0.872	*0.031*
Fe	*< 0.001*	0.912	*0.001*	0.837	*0.049*
Zn	*0.044*	0.912	0.981	0.662	*0.017*
Cu	*0.009*	0.362	0.981	0.872	0.538
Mn	*< 0.001*	0.912	*0.019*	0.639	0.497
Ni	*0.038*	0.912	0.981	0.662	0.770
Pb	0.665	0.912	0.759	0.639	0.497
Hg	0.059	0.912	0.981	0.662	0.538
Se	*0.002*	0.217	0.952	0.639	0.888
Cr	0.835	0.362	0.692	0.837	0.538
As	*0.001*	0.912	0.202	0.662	0.214
Cd	0.362	0.912	0.091	0.662	0.521

Full details of models are reported in Table S2. Each *P*_FDR_ is the most conservative estimate between the *P*_FDR_ from likelihood ratio tests and the FDR-corrected *P* values from the randomization procedure. Significant effects (*P*_FDR_ < 0.05) are reported in italic

**Fig. 3:**
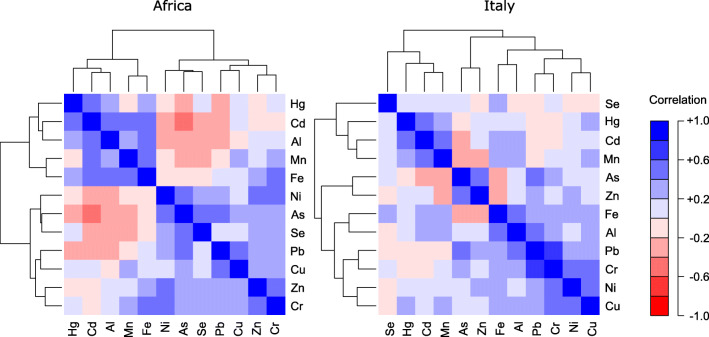
Heatmaps of correlations among trace element concentrations measured in barn swallow tail feathers grown in wintering grounds (Africa; R4nb) and breeding areas (Italy; R4br)

Besides differences in the elemental patterns of abundance between R4nb and R4br, differences in correlation patterns between trace elements were found (Fig. 3). In particular, in R4nb, two clusters of elements appeared. The first one includes Al, Fe, Mn, Hg and Cd, while the second includes Zn, Cu, Ni, Pb, Se, Cr and As. Conversely, the patterns of association among trace elements were less clear in R4br. This suggests that our barn swallows spent their non-breeding staging periods in two main different geographical areas, characterized by a different elemental composition. Barn swallows show migratory connectivity at continental scale, with two clusters of individuals wintering in Africa to the south and the north of approximately 5° S (Ambrosini et al. 2009). Individuals breeding in Italy generally spend the non-breeding staging period to the north of the equator, but geolocator tracking of individuals breeding in the same geographical area where we collected feathers revealed that some individuals arrive much further south, up to South Africa (Liechti et al. 2015). The observed patterns of trace element covariation may reflect these two groups of individuals. However, elemental composition of feathers differed also among roosts in South Africa (Szép et al. 2009), so the observed patterns may also reflect variability in the non-breeding staging sites at a smaller spatial scale. Unfortunately, we have no information on the actual non-breeding staging sites of the individuals whose feather we collected, so we cannot assess whether these patterns of covariation among trace element abundances actually reflect non-breeding staging area.

Al and Fe were the elements showing the wider difference in feather concentration between breeding and non-breeding staging areas (Table 1, Fig. 2). High levels of these elements in barn swallow feathers could be expected, considering that parent materials of African soils are rich in earth elements, including Al and Fe (Innangi et al. 2019). Accordingly, a similar pattern of trace elements was found in a previous study of the sand martin exploring the differences in the elemental composition of tail feathers grown in African non-breeding staging areas compared to those grown in European breeding ones (Szép et al. 2003, 2009). Such investigation showed that the concentration of ten elements, namely, Cd, Mn, Sr, Co, Fe, Li, Ti, V, Ba and Pb, was higher in feathers grown in non-breeding grounds, while the levels of Se, Zn, P and S were lower than those measured in European breeding areas (Szép et al. 2003). Interestingly, levels of Se and Zn were higher in Europe than in Africa in both the barn swallow and the sand martin (Szép et al. 2003). The low levels of Se and Zn measured in feathers grown in Africa suggest that the environmental levels of these trace elements were lower in the wintering grounds compared to the breeding quarters. In fact, Zn and Se are two of the main micronutrients that are deficient in soils of the Sub-Saharan Africa, where multiple deficiencies of up to five micronutrients are widespread (Berkhout et al. 2017). For instance, depletion of Se is common in soils of Sub-Saharan Africa because of soil erosion, leaching and volatilization through burning (Christophersen et al. 2013). The northern edge of the Sahel region in West Africa, the areas around the Congo basin (Eastern Africa) and Southern Africa are the regions more affected by the co-occurrence of micronutrient deficiencies (Berkhout et al. 2017). Considering that the non-breeding staging areas of the barn swallows breeding in colonies of Northern Italy and included in the present study were identified in Eastern Africa, specifically in Cameroon and its neighbouring countries (Liechti et al. 2015), we can suppose that low levels of Zn and Se accumulated in tail feathers grown in Africa reflected the depletion of these elements in the soils of these specific geographical areas.

### Sex-specific differences in trace element levels

In spite of differences in elemental fingerprint between wintering and breeding areas, no significant difference in the concentration of any element was detected according to sex or to the sex by feather growth location interaction both in multivariate (Table 2) and univariate analyses (Table 3). These results are consistent with previous studies of different passerine species, including the northern cardinals *Cardinalis cardinalis* and the great crested flycatchers *Myiarchus crinitus* (Cooper et al. 2017), the collared flycatcher (Óvári et al. 2018) and the Italian sparrow (Innangi et al. 2019), reporting that sex-specific differences were not observed for different trace elements accumulated in feathers. The lack of sex-specific differences in the elemental fingerprint of barn swallow feathers suggests that the bioaccumulation rates of trace elements are consistent between both the sexes (Cooper et al. 2017), probably because males and females use the habitats and exploit the same diet in both wintering and breeding areas (Møller 1994; Turner 2006; Liechti et al. 2015).

## Conclusions

The present study described the levels and the fingerprint of trace elements accumulated in tail feathers of the barn swallow. Trace elements were detected in measurable concentrations in tail feathers collected from all the individuals we captured in their breeding colonies. The most abundant elements were Al, Fe and Zn, while the levels of the most toxic elements, including Pd, Ni, Cd and Hg, were very low. Interestingly, the levels and the elemental fingerprint differed between feathers grown in the breeding and non-breeding staging areas, with overall higher levels in feathers grown in Africa than in Italy, with the exception of Zn and Se, suggesting different environmental sources of trace elements. Considering the occurrence and the potential toxicity of some trace elements, further research would be necessary to shed light on the elemental profile of wintering and breeding areas in order to investigate the sources and the accumulation pathways of trace elements in feathers of barn swallows. Moreover, these results further confirm that the trace element profile of feathers moulted in Africa may help identifying the areas where individuals spend the boreal winter.

## Supplementary Information

ESM 1 (PDF 31 kb)

## Data Availability

The datasets used and/or analysed during the current study are available from the corresponding author on reasonable request.
