# Supplementary material for: Trace elements fingerprint of feathers differs between breeding and non-breeding areas in an Afro-Palearctic migratory bird, the barn swallow (Hirundo rustica)
Source: Environ Sci Pollut Res Int. 2020 Nov 26;28(13):15828–37. doi: 10.1007/s11356-020-11597-z (PMC7969698; doi:10.1007/s11356-020-11597-z)
Supplement: Supplementary file 1 — (PDF 31 kb) [file 11356_2020_11597_MOESM1_ESM.pdf]

Supporting information

Trace elements fingerprint of feathers differs between breeding and non-breeding areas in an Afro-palearctic migratory bird, the barn swallow (*Hirundo rustica*)

Marco Parolini[1], Michela Sturini[2], Federica Maraschi[2], Antonella Profumo[2], Alessandra Costanzo[1], Manuela Caprioli[1], Diego Rubolini[1], Roberto Ambrosini[1*] and Luca Canova[2]

[1] *Department of Environmental Science and Policy, University of Milan, via Celoria 26, I-20133 Milan, Italy*

[2] *Department of Chemistry, University of Pavia, via Taramelli 12, I-27100 Pavia, Italy*

*Correspondence should be addressed to: Prof. Roberto Ambrosini (roberto.ambrosini@unimi.it)

Details on the randomization procedeure used in the statistical analyses

The randomization procedure used in this study to assess significance of univariate and multivariate models was aimed at accounting for the non-independence of data, inherent in the fact that two feathers were collected from the same individual and that different individuals bred in the same farm, and can thus share the same general ecological conditions, at least in the breeding ground. To this aim, we imposed a restriction in the randomization procedure whereby we randomly shuffled feather growth location within individuals, and sex between individuals of the same farm. To clarify this procedure, imagine that feathers A and B were collected from individual 1, which was a female and feather C and D from individual 2, which was a male and that individuals 1 and 2 bred in the same farm. Feathers A and C were grown in Africa, while B and D in Italy. The within individual randomization implied that if in a step of the randomization feather A and D were assigned to the group of feathers grown in Africa, feathers B and C were necessarily assigned to the group of feathers grown in Italy. This procedure assured that at each run of the randomization procedure, one feather of each individual was always assigned to the group of feathers grown in Italy and one to that of the feathers grown in Africa. In contrast, the randomization of sexes between individuals implied that if in a run we assigned individual 1 to the males group, we assigned both feathers A and B to this group.

Table S1: Detection and quantification limits of trace elements. Method detection and quantification limits (MDLs, MQLs, respectively), as well as instrumental detection and quantification limits (IDLs, IQLs, respectively) are reported.

	IDL (µg/L)	IQL (µg/L)	MDL (mg/kg)	MQL (mg/kg)
As	0.06	0.2	0.03	0.1
Al	0.06	0.2	0.03	0.1
Cd	0.03	0.1	0.02	0.05
Cu	0.01	0.03	0.01	0.02
Zn	0.05	0.15	0.03	0.1
Hg	0.06	0.2	0.03	0.1
Mn	0.01	0.03	0.01	0.02
Pb	0.01	0.03	0.01	0.02
Cr	0.05	0.15	0.03	0.1
Ni	0.01	0.03	0.01	0.02
Fe	0.6	1.8	0.3	0.9
Se	0.2	0.6	0.1	0.3

Table S2: Full report of the results of linear mixed models used for univariate analyses of elemental abundance summarized in Table 3 of the main text. First part of each table reports information on the fixed effects, the second part on the random effects. L.R. are the values of likelihood ratio tests ; P_{FDR} is the most conservative estimate between the P-values corrected with the False Discovery Rate procedure from Likelihod Ratio Tests and the FDR-corrected P-values from the randomization procedure (also reported in Table 3 in the main text); $\tau_{00\ ID}$ and $\tau_{00\ farm}$ are the square roots of variance components (standard deviations) for, respectively, individual identity and farm identity; $\tau_{11\ ID.Location}$ is the square root of variance component of feather growth location (dichotomic factor) entered as a random slope within individual; $\rho_{01\ ID}$ is the correlation coefficient between $\tau_{00\ ID}$ and $\tau_{11\ ID.Location}$. In all models, number of observations is 118, number of individuals is 59, and number of farms is 5.

	**Al**			
**Predictors**	**Estimates**	**S.E.**	**L.R.**	**P_{FDR}**
Intercept	176.19	43.38		
Location	-61.73	46.40	18.195	0.035
Sex	79.57	58.20	0.340	0.912
Age	326.62	98.65	5.065	0.063
Location × Sex	-77.16	62.16	1.541	0.872
Location × Age	-287.15	102.66	7.823	0.031
**Random Effects**				
	S.D.	$\rho_{01\ ID}$		
$\tau_{00\ ID}$	0.000			
$\tau_{11\ ID.Location}$	59.370	-0.84		
$\tau_{00\ farm}$	0.003			

Table S2 (continued)

Fe				
**Predictors**	**Estimates**	**S.E.**	**L.R.**	**P_{FDR}**
Intercept	149.38	9.58		
Location	-47.60	11.78	57.722	<0.001
Sex	9.68	12.78	0.300	0.912
Age	93.11	21.44	26.323	0.001
Location × Sex	-8.39	15.38	0.297	0.837
Location × Age	-58.60	23.37	6.286	0.049
**Random Effects**				
	S.D.	$\rho_{01\ ID}$		
$\tau_{00\ ID}$	0.00			
$\tau_{11\ ID.Location}$	32.600	1.00		
$\tau_{00\ farm}$	0.083			

Zn				
**Predictors**	**Estimates**	**S.E.**	**L.R.**	**P_{FDR}**
Intercept	136.83	4.16		
Location	24.86	6.60	4.537	0.044
Sex	-5.44	4.82	0.870	0.912
Age	8.93	5.34	0.002	0.981
Location × Sex	4.10	6.14	0.445	0.662
Location × Age	-28.86	6.96	17.205	0.017
**Random Effects**				
	S.D.	$\rho_{01\ ID}$		
$\tau_{00\ ID}$	18.251			
$\tau_{11\ ID.Location}$	10.612	-0.97		
$\tau_{00\ farm}$	3.521			

Table S2 (continued)

	Cu			
**Predictors**	**Estimates**	**S.E.**	**L.R.**	**P_{FDR}**
Intercept	11.45	0.60		
Location	-1.75	0.73	17.953	0.009
Sex	-1.20	0.62	4.103	0.362
Age	0.30	0.62	0.001	0.981
Location × Sex	0.54	0.89	0.364	0.872
Location × Age	-0.71	0.87	0.666	0.538
**Random Effects**				
	S.D.	$\rho_{01\ ID}$		
$\tau_{00\ ID}$	0.370			
$\tau_{11\ ID.Location}$	0.962	0.85		
$\tau_{00\ farm}$	0.712			

	Mn			
**Predictors**	**Estimates**	**S.E.**	**L.R.**	**P_{FDR}**
Intercept	9.48	0.67		
Location	-7.46	0.66	271.476	<0.001
Sex	-0.92	0.80	0.092	0.912
Age	3.13	1.32	8.699	0.019
Location × Sex	0.99	0.88	1.284	0.639
Location × Age	-1.80	1.44	1.552	0.497
**Random Effects**				
	S.D.	$\rho_{01\ ID}$		
$\tau_{00\ ID}$	0.000			
$\tau_{11\ ID.Location}$	0.000	0.75		
$\tau_{00\ farm}$	0.702			

Table S2 (continued)

	Ni			
**Predictors**	**Estimates**	**S.E.**	**L.R.**	**P_{FDR}**
Intercept	5.08	0.38		
Location	-1.02	0.46	7.646	0.038
Sex	-0.18	0.39	0.101	0.912
Age	-0.16	0.40	0.016	0.981
Location × Sex	0.39	0.54	0.526	0.662
Location × Age	0.21	0.55	0.143	0.770
**Random Effects**				
	S.D.	$\rho_{01\ ID}$		
$\tau_{00\ ID}$	1.234			
$\tau_{11\ ID.Location}$	1.663	-0.96		
$\tau_{00\ farm}$	0.445			

	Pb			
**Predictors**	**Estimates**	**S.E.**	**L.R.**	**P_{FDR}**
Intercept	4.44	0.52		
Location	-0.51	0.37	0.319	0.665
Sex	-0.47	0.31	1.137	0.912
Age	-0.74	0.34	3.833	0.759
Location × Sex	0.57	0.49	1.315	0.639
Location × Age	1.07	1.07	1.006	0.497
**Random Effects**				
	S.D.	$\rho_{01\ ID}$		
$\tau_{00\ ID}$	1.158			
$\tau_{11\ ID.Location}$	1.176	-1.00		
$\tau_{00\ farm}$	1.019			

Table S2 (continued)

Hg				
**Predictors**	**Estimates**	**S.E.**	**L.R.**	$\mathbf{P_{FDR}}$
Intercept	3.17	0.56		
Location	-1.03	0.51	8.120	0.059
Sex	-0.57	0.67	0.170	0.912
Age	0.69	0.81	0.009	0.981
Location × Sex	0.50	0.66	0.584	0.662
Location × Age	-0.68	0.79	0.741	0.538
**Random Effects**				
	S.D.	$\rho_{01\ ID}$		
$\tau_{00\ ID}$	0.916			
$\tau_{11\ ID.Location}$	0.706	-0.69		
$\tau_{00\ farm}$	0.488			

Se				
**Predictors**	**Estimates**	**S.E.**	**L.R.**	$\mathbf{P_{FDR}}$
Intercept	1.45	0.27		
Location	1.05	0.28	18.998	0.002
Sex	-0.05	0.26	11.303	0.217
Age	0.03	0.34	0.240	0.952
Location × Sex	-0.57	0.36	2.506	0.639
Location × Age	0.07	0.44	0.025	0.888
**Random Effects**				
	S.D.	$\rho_{01\ ID}$		
$\tau_{00\ ID}$	0.496			
$\tau_{11\ ID.Location}$	1.020	-0.91		
$\tau_{00\ farm}$	0.417			

Table S2 (continued)

Cr				
**Predictors**	**Estimates**	**S.E.**	**L.R.**	**P_{FDR}**
Intercept	1.16	0.16		
Location	-0.09	0.20	0.686	0.835
Sex	-0.21	0.11	4.881	0.362
Age	0.30	0.12	6.317	0.692
Location × Sex	0.11	0.26	0.173	0.837
Location × Age	-0.23	0.28	0.677	0.538
**Random Effects**				
	S.D.	$\rho_{01\ ID}$		
$\tau_{00\ ID}$	0.203			
$\tau_{11\ ID.Location}$	0.835	-0.85		
$\tau_{00\ farm}$	0.301			

As				
**Predictors**	**Estimates**	**S.E.**	**L.R.**	**P_{FDR}**
Intercept	0.36	0.02		
Location	0.08	0.02	15.934	0.001
Sex	0.01	0.02	0.046	0.912
Age	-0.03	0.02	2.443	0.202
Location × Sex	-0.02	0.03	0.859	0.662
Location × Age	-0.05	0.03	3.293	0.214
**Random Effects**				
	S.D.	$\rho_{01\ ID}$		
$\tau_{00\ ID}$	0.054			
$\tau_{11\ ID.Location}$	0.063	0.46		
$\tau_{00\ farm}$	0.040			

Table S2 (continued)

	Cd			
**Predictors**	**Estimates**	**S.E.**	**L.R**	**P_{FDR}**
Intercept	0.02	0.01		
Location	0.00	0.01	3.646	0.362
Sex	-0.01	0.01	0.437	0.912
Age	0.01	0.01	4.929	0.091
Sex	0.01	0.01	0.921	0.662
Location × Age	0.01	0.01	1.242	0.512
**Random Effects**				
	S.D.	$\rho_{01\ ID}$		
$\tau_{00\ ID}$	0.014			
$\tau_{11\ ID.Location}$	0.035	0.34		
$\tau_{00\ farm}$	0.024			
